# Gastric intramural metastasis caused by needle tract seeding after preoperative fine needle aspiration for pancreatic body cancer subsequently resected by total pancreatectomy: a case report and literature review

**DOI:** 10.1186/s12957-023-02914-0

**Published:** 2023-02-13

**Authors:** Eiji Yoshida, Yasutoshi Kimura, Takuro Kyuno, Ryoko Kawagishi, Kei Sato, Tsuyoshi Kono, Takehiro Chiba, Toshimoto Kimura, Hitoshi Yonezawa, Osamu Funato, Makoto Kobayashi, Yoshiko Keira, Kazunori Onuma, Hiroyuki Inoue, Akinori Takagane, Ichiro Takemasa

**Affiliations:** 1 Department of Surgery, Hakodate Goryoukaku Hospital, 38-3, goryoukaku-cho, Hakodate, Hokaido 040-8611 Japan; 2 Department of Surgery, Surgical Oncology and Science, Sapporo Medical University, S-1, W-16, Chuo-ku, Sapporo, Hokkaido 060-8543 Japan; 3 Department of Surgical Pathology, Hakodate Goryoukaku Hospital, 38-3, goryoukaku-cho, Hakodate, Hokaido 040-8611 Japan; 4 Department of Gastroenterology and Hepatology, Hakodate Goryoukaku Hospital, 38-3, goryoukaku-cho, Hakodate, Hokaido 040-8611 Japan

**Keywords:** Needle tract seeding, Gastric metastasis, Pancreatic cancer, Total pancreatectomy

## Abstract

**Background:**

Recently, there has been an increase in the number of reports of needle tract seeding (NTS) of tumor cells after a biopsy as one of the adverse events related to endoscopic ultrasonography-guided fine needle aspiration (EUS-FNA). In most of the previously reported cases of NTS in pancreatic cancer, distal pancreatectomy was performed as the initial surgery, following which metachronous metastasis was discovered in the gastric wall, whose localization matched the puncture route of the EUS-FNA. We report a case of early metastasis from pancreatic cancer in the gastric wall, which was postulated to be caused by NTS. Our patient underwent a total pancreatectomy (TP), and the NTS was resected synchronously.

**Case presentation:**

A 70-year-old woman with a diagnosis of pancreatic head-body-tail cancer presented to our department for surgery. Transgastric EUS-FNA and biopsy established the histological diagnosis in her case. We administered neoadjuvant chemotherapy (NAC) to the patient and performed a TP. Histopathological and immunohistochemical examination subsequently confirmed the diagnosis of pT3N1aM1 pancreatic adenocarcinoma and its gastric metastasis, which was caused by NTS. It is postulated that the tumor cells of NTS had progressed to develop the metastatic lesion in the gastric wall during the NAC period. This was also resected during the initial surgery. The patient developed an early postoperative recurrence in the peritoneum 8 months after the surgery.

**Conclusion:**

In pancreatic head cancer cases, the puncture route is often included in the resection area of radical surgery, and NTS is seldom considered as a potential clinical problem. However, NTS can progress rapidly and may be associated with early recurrence of malignancy. Therefore, when transgastrointestinal puncture is performed for the diagnosis of pancreatic cancer, the treatment strategy should be established considering the potential development of NTS.

## Background

Recently, neoadjuvant therapy (NAT) has gained popularity as a common preoperative intervention for pancreatic cancer, thus highlighting the importance of establishing a pathological diagnosis before starting initial treatment [1, 2].

Several methods are available and can be utilized to reach a pathological diagnosis of pancreatic cancer, such as endoscopic ultrasound-guided fine needle aspiration (EUS-FNA), which is a well-established and commonly utilized method [3, 4].

Over the years, preoperative EUS-FNA has risen due to increased demand, with consequently augmented reports of its adverse effects [5, 6]. Needle tract seeding (NTS) is also a resultant adverse event, a phenomenon in which tumor cells are implanted in the puncture route during EUS-FNA. Most reports have demonstrated NTS to be a metachronous metastasis developing after distal pancreatectomy (DP): cancer cells seeded in the needle tract and left in situ after the initial surgery might require some time to form a mass within the gastric wall. Therefore, it is rare for the NTS to be resected simultaneously as the primary tumor [7–9]. We encountered a case in which a total pancreatectomy was performed after NAT for a pancreatic cancer diagnosed by FNA, whose resected specimen revealed the presence of NTS. We herein report this rare case since its clinical course may be significant in suggesting a relationship between NTS and early postoperative recurrence of malignancy.

## Case presentation

A 70-year-old woman presented to the referring hospital with a complaint of back discomfort. Her carbohydrate antigen 19-9 (CA19-9) levels were discovered to be elevated on subsequent testing, and the attending physician suspected a diagnosis of pancreatic cancer. The patient was referred to our hospital for further investigation.

The patient reported a normal appetite, despite a weight loss of 3 kg in the last month. On physical examination, her abdomen was soft, and no mass was felt during palpation. Her medical history included hypertension, hyperlipemia, and diabetes, treated by an antihypertensive agent, 3-hydroxymethyl-3-methylglutaryl coenzyme A reductase inhibitor, and an oral hypoglycemic agent, respectively. The patient had undergone two cesarean sections at 42 and 44 years. Her laboratory results demonstrated elevated hemoglobin A1c (9.4%) as well as tumor markers, including carbohydrate antigen 19−9 (CA19−9) (3696 U/mL), DUPAN-2 (1600 U/mL), and SPan-1 (220 U/mL). Bilirubin levels were normal.

**Fig. 1:**
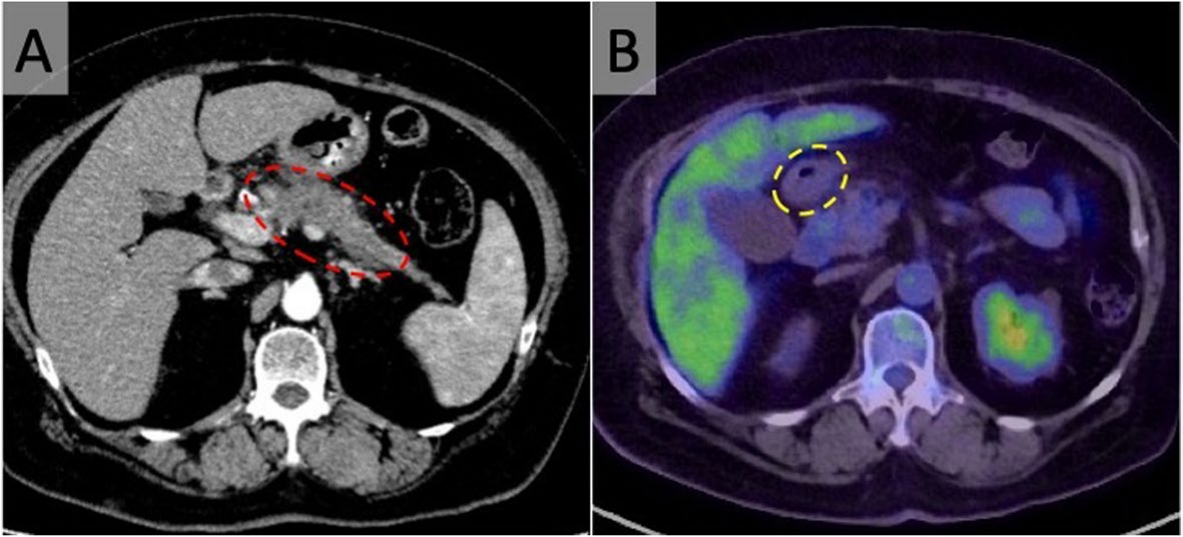
Imaging findings. **A** Abdominal axial image of computed tomography (CT) shows a tumor with poor contrast in the pancreatic head-body-tail (red dotted line). **B** Positron emission tomography-CT shows no evidence of distant metastasis, including gastric metastasis. Yellow dotted line indicates the pyloric ring

Enhanced computed tomography (CT) revealed a 70-mm pancreatic head-body-tail tumor with poor enhancement in the early phase (Fig. 1A). The tumor exhibited invasion and attachment to the vessels, such as the splenic artery and vein (SPA/SPV), superior mesenteric vein (SMV), and gastroduodenal artery (GDA). No metastases to distant organs were apparent, although the suprapancreatic lymph node was suspected of metastatic disease. Transgastric endoscopic ultrasonography-guided fine needle aspiration was performed using a 22-gauge needle (EZ Shot 3 Plus, Olympus, Tokyo, Japan) with two negative pressure punctures through the same tract. Biopsy samples were obtained from the tumor. Histopathological analysis of the biopsy sample revealed adenocarcinoma. She was diagnosed with cT3N1M0 pancreatic cancer (Union for International Cancer Control [UICC]-TNM classification, eighth edition) [10]. According to the National Comprehensive Cancer Network (NCCN) guidelines, the tumor was resectable [11]. In this case, total pancreatectomy (TP) was deemed necessary to achieve a curative resection, and NAT was introduced prior to the surgery. Good glycemic control, important for surgical conditioning, was mandated in parallel with NAT. The NAT regimen included gemcitabine plus S-1 (an oral fluoropyrimidine agent containing tegafur, gimeracil, and oteracil potassium) therapy, which consisted of intravenous gemcitabine at a dose of 1000 mg/m^2^ on days 1 and 8 and oral S-1 at a dose of 60 mg twice daily on days 1-14 of a 21-day cycle; two courses of NAT were administered. After completing the preoperative treatment, we confirmed that there was neither progression of the tumor nor development of any distant metastases by utilizing multiple modalities, such as CT, enhanced magnetic resonance imaging (MRI), and positron emission tomography (PET)-CT (Fig. 1B). CA19-9 levels had markedly decreased but still had not normalized (713 U/mL). Concomitantly, TP was performed.

### Surgical procedure and intraoperative details

A median incision was placed in the upper abdomen. The left retroperitoneal space was accessed from the level of the proximal jejunum. The part of the mesocolon covering the tumor and the retroperitoneal fat tissue were dissected along with the tumor. The Kocher maneuver was performed, and the omental bursa was released open to expose the anterior surface of the pancreatic head and duodenum. The suprapancreatic lymph node was suspected to be metastatic but had not infiltrated the common hepatic artery (CHA). Therefore, it was dissected away from the CHA. We could encircle the roots of the SPA and GDA sufficiently away from tumor invasion; hence, we judged this case to be resectable. Subsequently, we divided the bile duct, SPA, and GDA. The gastric antrum was divided at 30 mm proximal to the pyloric ring. Afterward, the nerve plexus of the pancreatic head was isolated. Tumor invasion of the SPV confluence was suspected when dissecting the portal vein (PV) surface. We performed PV resection (wedge style) with SPV and subsequent reconstruction. We dissected the pancreatic body-tail-spleen from the retroperitoneum and removed the specimen. The operation lasted 591 min, and the blood loss was estimated to be 920 ml. No intraoperative blood transfusion was required.

### Pathological findings on the resected specimen

**Fig. 2:**
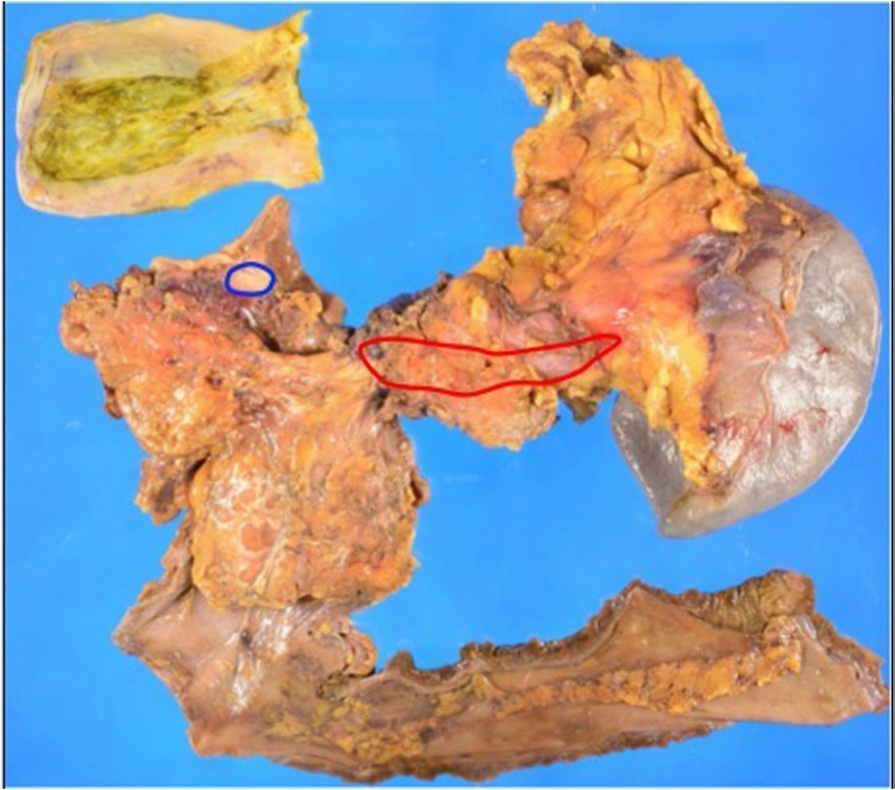
Pathological findings. Tumor mapping on the divided surface of the specimens showing an invasive tumor with a 70-mm diameter in the pancreatic body-tail (red line) and another tumor with an 8-mm diameter in the gastric wall (blue line)

**Fig. 3:**
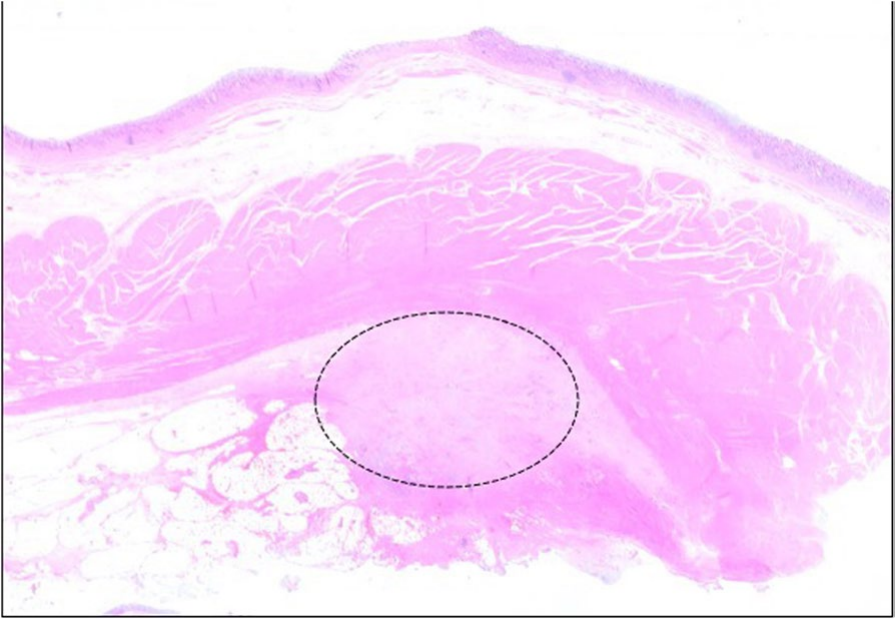
Histopathological findings of gastric wall (hematoxylin and eosin staining, loupe image). Histopathological examination reveals growth of cancer in gastric wall (black dotted line). It extends from the subserosal layer to proper muscular layer, with no exposure to the mucosal surface

**Fig. 4:**
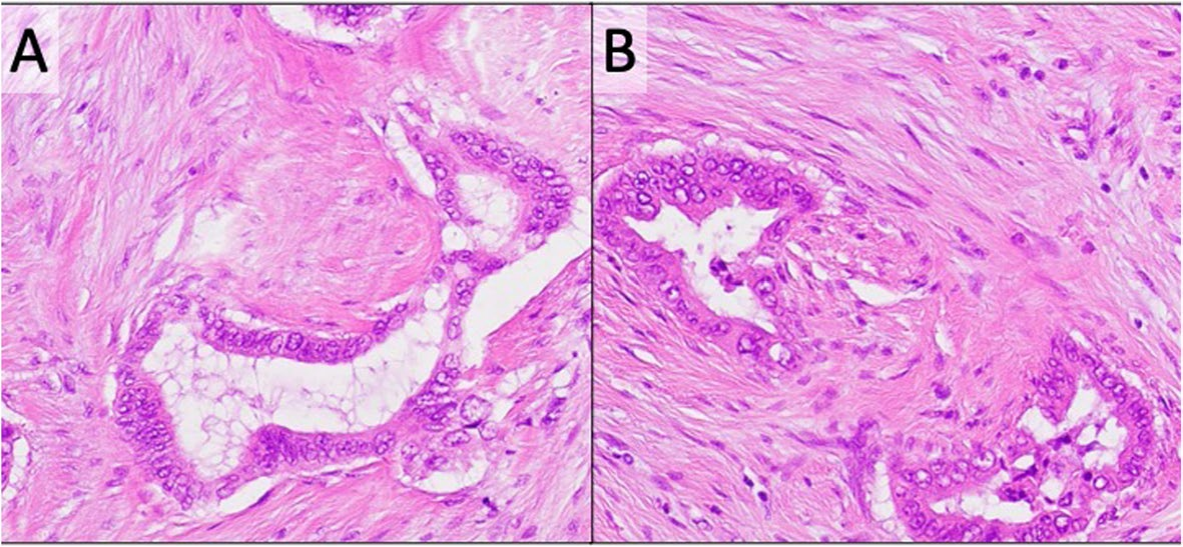
Comparison of histopathological findings (hematoxylin and eosin staining, × 20). **A** Histopathological examination shows moderately differentiated adenocarcinoma of pancreas. **B** The gastric wall lesion is also histologically moderately differentiated adenocarcinoma, similar to the primary pancreatic cancer.

**Fig. 5:**
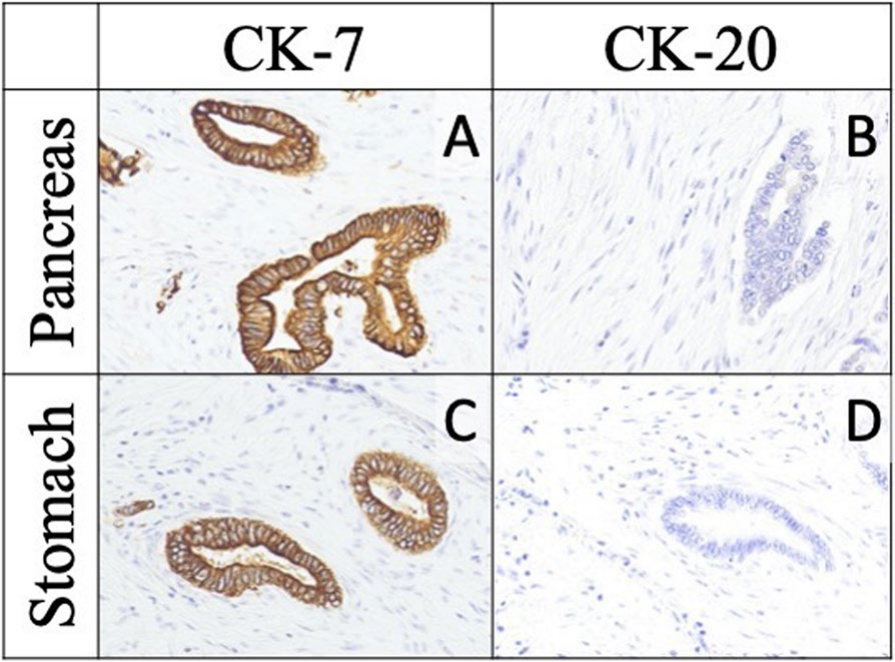
Comparison of immunohistochemical findings (immunohistochemical staining for CK-7 and CK-20, × 20). Adenocarcinoma of gastric wall is positive for CK-7 and negative for CK-20; this is similar to the findings of the primary pancreatic adenocarcinoma

The resected specimen demonstrated pancreatic body cancer, with an additional adenocarcinoma lesion in the stomach wall (Fig. 2). The gastric tumor location extended from the subserosal to the proper muscular layer, with no exposure to the mucosal surface (Fig. 3). Considering this peculiar localization and its histological similarities to the primary pancreatic tumor, the stomach tumor was also suggested to be a pancreatic cancer metastasis (Fig. 4). Immunohistochemical studies, using anti-cytokeratin 7 and 20 monoclonal antibodies, demonstrated positivity for cytokeratin 7 and negativity for cytokeratin 20 in both pancreatic and gastric tumors. The expression of these cytokeratin subtypes showed the same pattern in both the gastric and pancreatic tumors, providing evidence that the gastric tumor was a metastasis from the pancreatic cancer (Fig. 5). Moreover, peritoneal washing cytology was found to be positive. Thus, the pathological diagnosis established was pancreatic adenocarcinoma, pT3N1aM1. The cancer was classified as Stage IV based on the eighth edition of the UICC-TNM classification [10]. The pathological treatment effect of NAT was graded as IIa based on the Evans classification [12].

### Postoperative course

Postoperatively, there were no complications, and the patient could resume dietary intake four days post-surgery. She was discharged on the 24th postoperative day after being monitored for glycemic control by a diabetologist. The patient was initiated on adjuvant chemotherapy with S-1 orally, but she had an initial recurrence in the peritoneum 8 months after the surgery. Subsequently, she was administered systemic chemotherapy and was alive at the last checkup, 4 months after the recurrence.

## Discussion

EUS-FNA is an important method for confirming the pathological diagnosis of pancreatic cancer. This is essential before initiating treatment. Adverse events such as bleeding and pancreatitis are only seen in 1.7% of the cases with EUS-FNA; hence, it is recognized as a relatively safe method [13]. However, the number of reports on NTS have been increasing in recent years.

There is no formal criterion for the diagnosis of NTS, and it is determined completely based on its similarity in histological characteristics to the primary tumor, its localization in a particular layer, its presence consistent with the puncture route, and the fact that it is a metastasis in the relevant organ only. While proving that the location of the tumor is consistent with the route of EUS-FNA is a reliable indicator for the diagnosis of NTS, this is often difficult to establish in practice. In our case, the diagnosis of NTS was made based on a solitary tumor that extended from the subserosal to the proper muscular layer in the stomach and demonstrated similar histological findings, as well as the same immunohistochemical pattern as the primary pancreatic tumor. The tumor was located on the posterior wall of the stomach, near the pyloric ring, and was consistent with the EUS-FNA route.

The incidence of soft tissue metastasis caused by larger diameter needles is greater than that of smaller ones [14]. We often use the 22-gauge needle, which is not thick. Furthermore, Sakamoto et al. reported that it cannot be concluded that a lower number of punctures are directly related to the prevention of NTS [15]. In our case, NTS occurred although only two punctures were performed. Using smaller needles or avoiding multiple punctures of the same needle may preclude NTS. However, when transgastric puncture was performed, it is necessary to implement subsequent treatment with the concern of NTS regardless of its procedure.

Through a review of case reports in the English literature utilizing the PubMed electronic database following input of the terms “needle tract seeding”, “pancreatic cancer”, and “EUS-FNA”, we identified 18 cases of NTS on the gastric wall caused by EUS-FNA of pancreatic cancer (Table 1) [7–9, 16–28]. In these cases, the tumor was located in the pancreatic body-tail in all the cases, and 94.1% of the cases undergoing surgery implemented a DP. There was no report of NTS related to pancreatic head cancer. This could be explained by the fact that the puncture route is usually resected en-bloc with the primary tumor in cases of pancreatic head cancer. However, in the case of transgastric EUS-FNA conducted for pancreatic head-neck cancer, there is a concern for NTS due to the residual puncture route. It usually takes some time for the NTS lesions to appear, which is another probable reason why NTS in pancreatic head cancer is rarely reported. In our case, transgastric EUS-FNA was performed for the pancreatic body cancer extending to the neck. While the puncture route was excised by subsequent surgery, the NTS was evident in the resected specimen owing to its early occurrence. According to this literature review, the median interval from pancreatic resection to detection of gastric metastasis was 14 months (range, 0–42 months). Notably, in four cases, the NTS lesion was resected at the same time as the primary surgery, and three of these four cases had undergone NAT, as in our case. This suggests that metastases developed and became apparent during the NAT period. In our case, NAT was administered, and there was a 3-month interval between EUS-FNA and radical surgery. Previous studies on different carcinomas reported that the local treatment effects of adjuvant therapy cannot be disregarded in potentially eradicating NTS [29]. In addition, a recent study of pancreatic cancer found that failure of CA19-9 to normalize from preoperative levels by the time of surgery is a predictor for early postoperative recurrence [30]. For our patient, the failure of tumor markers to normalize probably suggested inadequate effectiveness of NAT. Therefore, this may be associated with the rapidly developed NTS and the early postoperative recurrence in this case.

**Table 1:** Literature review of cases of NTS on gastric wall caused by EUS-FNA of pancreatic cancer

Factor	Subject	Number of cases
		(*N* = 18)
Age	Year, median (range)	69 (50–87)
Gender	Male/female	7/11
Location of cancer	Pb/Pt/Pbt/none described^a^	11/5/1/1
Primary treatment	DP/CP/RT^b^	16/1/1
Detection device	EGD/PET-CT/CT/palpation/EUS	7/4/4/2/1
Interval from surgery	Month, median (range)	14 (0–42)
Neoadjuvant therapy	Yes/no	3/15
Treatment for metastasis	pG/TG/DG/chemotherapy/no/none described^c^	11/1/1/1/1/3

^a^*Pb* pancreatic body, *Pt* pancreatic tail, *Pbt* pancreatic body-tail

^b^*RT* radiation therapy

^c^*pG* partial gastrectomy, *TG* total gastrectomy, *DG* distal gastrectomy

The long-term prognostic impact of NTS and the association between NTS and late distant metastases remain unclear [31]. Retrospective studies investigating the effects of the preoperative use of EUS-FNA for pancreatic cancer indicated no differences in long-term outcomes between the EUS-FNA group and the non–EUS-FNA group. They also did not demonstrate any obvious negative effects of preoperative EUS-FNA on recurrence-free survival and overall survival [31–37]. In contrast, a study reported an unexpectedly high risk of NTS following EUS-FNA, and another study reported concerns about the prognostic impact of EUS-FNA on resected pancreatic cancer cases [38, 39].

Katanuma et al. reported that NTS may lead to distant metastasis by facilitating the spread of tumor cells via the lymphatic vessels [19]. Further, Nakatsubo et al. noted that these metastases may occur due to the adherence of tumor cells to the blood and lymphatic vessels [40]. Although there is no evidence that NTS causes recurrence in the peritoneum, a previous study reported that cancer cells were often found in the gastrointestinal luminal fluid following EUS-FNA in patients with pancreatic tumors [39]. Therefore, these cells might have been translocated from extraluminal sites into the gastrointestinal tract and intervening tissues. In other words, cancer cells may transfer from the puncture route to the peritoneal cavity, leading to recurrence in the peritoneum [39, 40]. Furthermore, a recent study found that the ratio of peritoneal recurrence tended to be greater in the cases in which peritoneal washing cytology is positive [41]. According to these findings, a positive cytology status in a patient who underwent EUS-FNA, such as our case, may indicate a risk of NTS, including peritoneal dissemination.

In our case, no metastases in other organs were discovered on preoperative imaging examinations, and the gastric metastasis was suggested to be solitary. However, the patient developed early postoperative recurrence in the peritoneum, suggesting NTS might have some oncological impact. It is desirable to conduct large-scale prospective studies to reach a consensus on this clinical question that currently has conflicting answers.

Even if NTS is not proven to be related to the prognosis, early diagnosis and resection might be effective for treating NTS as previously reported. It is also important to remove the needle tract en-bloc with pancreatectomy [22, 23, 32]. In other words, regarding cases that underwent transgastric EUS-FNA, it might be better to set a line of gastric transection that ensures the needle tract is included in the resection area in head cases and add a partial gastrectomy that includes needle tract resection in body-tail cases. According to this literature review, NTS was diagnosed through esophagogastroduodenoscopy (EGD) in 38.9% of the cases. Hence, postoperative routine EGD for regular follow-ups may be useful for detecting NTS in cases where the needle tract remains. However, microscopic NTS such as that detected in our case often exhibits no findings on the gastric mucosa, so regular follow-up with multiple imaging modalities is also important.

Conversely, if NTS is proven to have an impact on prognosis, EUS-FNA carries the risk of NTS, which is a rare but serious complication. Hence, it is necessary to identify pancreatic cancer cases where EUS-FNA should or should not be performed, and this requires further research. Alternatively, on a case-by-case basis, it might be useful to select a method that directly captures cancer cells without passing through the gastrointestinal tract, such as pancreatic duct biopsy or cytological diagnosis.

The treatment of pancreatic cancer has developed significantly in recent years, and recommendations are changing. To put precision medicine into practice in the future, biopsy at diagnosis will become increasingly necessary and important. Furthermore, we should recognize the latent risk of serious adverse events, such as NTS, which may be an adverse prognostic factor.

## Conclusion

We reported the case of pancreatic body cancer coexistent with rapidly developed NTS. Furthermore, this case with an early recurrence has a great impact suggesting NTS is not merely a local recurrence but is associated with poor prognosis or distant metastasis. NTS might potentially occur in all pancreatic cancers, even in the head of the pancreas. Consequently, NTS should be considered in the treatment strategy for pancreatic cancer.

## Data Availability

The dataset supporting the conclusions of this article is included within the article.

## Abbreviations

CA19-9: Carbohydrate antigen 19-9
CHA: Common hepatic artery
CT: Computed tomography
DP: Distal pancreatectomy
EGD: Esophagogastroduodenoscopy
EUS-FNA: Endoscopic ultrasonography-guided fine needle aspiration
GDA: Gastroduodenal artery
MRI: Magnetic resonance imaging
NAT: Neoadjuvant therapy
NCCN: National Comprehensive Cancer Network
NTS: Needle tract seeding
PET: Positron emission tomography
PV: Portal vein
SMV: Superior mesenteric vein
SPA/SPV: Splenic artery/splenic vein
TP: Total pancreatectomy
UICC: Union for International Cancer Control
